# Intravenous Leiomyosarcoma of the Lower Extremity: As Peripheral as It Gets

**DOI:** 10.7759/cureus.13073

**Published:** 2021-02-02

**Authors:** Levent F Umur, Selami Cakmak, Mehmet Isyar, Hamdi Tokoz

**Affiliations:** 1 Orthopaedics and Traumatology, Acibadem Kadikoy Hospital, Istanbul, TUR; 2 Cardiovascular Surgery, Acibadem Kadikoy Hospital, Istanbul, TUR

**Keywords:** intravenous leiomyosarcoma, sarcoma surgery

## Abstract

Leiomyosarcomas of the vascular system (vLMSs) are rare tumors that commonly originate from large proximal and central veins. Pancreatic metastasis is rare for sarcomas, and surgical excision with large margins is the treatment of choice. We present a case of a 32-year-old female with primary vLMS originating from the distal crural veins and local invasion of the fibula. A prior open biopsy site was suboptimal. The patient was treated with neoadjuvant chemotherapy and radiotherapy, followed by surgery. The follow-up radiological imaging showed pancreatic head metastasis, which is also an extremely rare site for vLMS.

## Introduction

Leiomyosarcomas of the vascular system (vLMSs) are rare tumors that commonly originate from large veins. Distally originated tumors are very rare. Leiomyosarcomas represent about 5-7% of all soft tissue sarcomas, and vLMSs represent only 2% of all these neoplasia. Bones and lungs are the most frequent metastatic sites of sarcomas. The pancreas is a very rare metastatic site for vLMSs as well as other sarcomas. The mean age for leiomyosarcomas is the sixth and seventh decades, and patients younger than 50 are rare.

The decision regarding the biopsy site and the tract is crucial for general bone and soft tissue malignancy management. It is preferable for the biopsy to be done by the center that is planning the surgical procedure. The biopsy should be accepted as the last stage of diagnosis and the first stage of treatment.

We present a case of a 32-year-old female with primary vLMS originating from the distal peroneal vein and local invasion of the fibula.

## Case presentation

**Figure 1:**
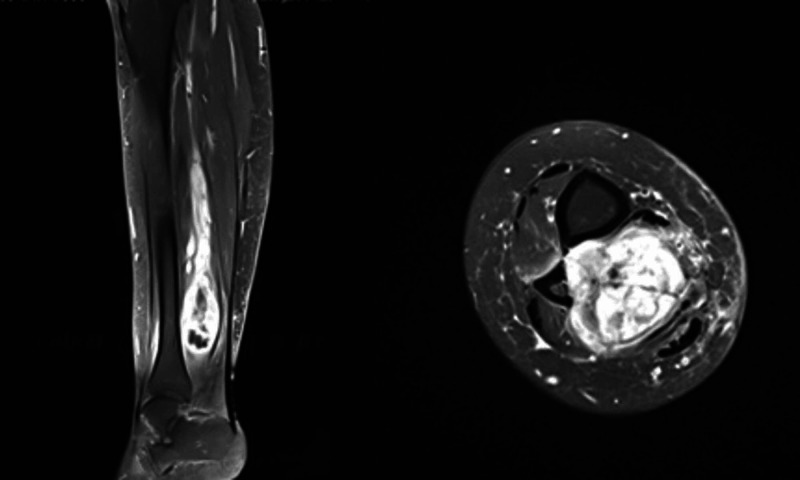
Sagittal and axial MRI views of the mass

A 32-year-old female patient had a history of leg pain that started in March 2018. After multiple conservative treatment cycles, Doppler ultrasound (USG) and computed tomography (CT) scans were performed due to persistent pain. A soft tissue mass was detected in the distal cruris, and an open biopsy from the posteromedial aspect of the distal cruris was performed in February 2019. The pathology report indicated biphasic synovial sarcoma, and the patient was referred to our institution with that diagnosis. The pathologic samples were reevaluated and reported as spindle cell sarcoma (French Federation of Cancer Centers Sarcoma Group histologic grade 3). Magnetic resonance imaging (MRI) showed a tissue mass measuring 4.82 x 4.16 x 20 cm in the mid-to-distal third of the posterior crural compartment with cortical destruction of the distal fibula just proximal to the ankle (Figure 1). The peroneal artery was inside the mass, and the posterior tibial artery was alongside the mass. The mass was compressing the distal popliteal vein. CT scans of the thorax and abdomen and positron emission tomography (PET)-CT showed no evidence of metastasis. Neo-adjuvant chemotherapy (ifosfamide plus etoposide) and radiotherapy were started. The chemotherapy doses were decreased after the patient experienced a generalized tonic-clonic seizure, and the anti-epileptic therapy dosage was increased. Acute deep vein thrombosis (DVT) occurred during the second chemotherapy cycle. After the third cycle, a control MRI showed insignificant downsizing of the mass and large necrosis sites, so surgery was scheduled.

**Figure 2:**
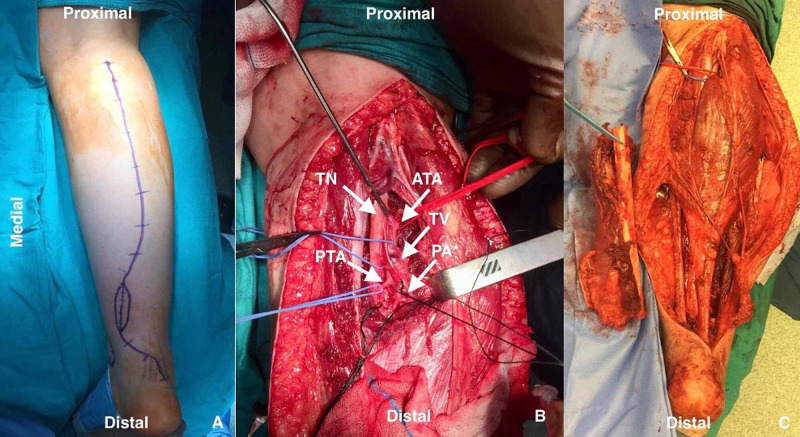
Intraoperative views (A) A lazy S incision was planned to ease the approach as well as the resection of the biopsy site; (B) The crural arteries were dissected and the peroneal artery sacrificed; (C) Resected specimen alongside the surgical site ATA: anterior tibial artery; PTA: posterior tibial artery: PA: peroneal artery; TN: tibial nerve; TV: tibial vein

A suboptimal posterior approach was utilized due to the position of the existing open biopsy site (Figure 2A). The crural arteries were dissected and the peroneal artery sacrificed (Figure 2B). The approach was carried out in a medial-to-lateral manner. The posterior tibial artery was dissected and the adventitia left on the mass. Frozen-section pathologic consultation showed no malignant cells. The tibial periosteum was left on the tumor and the 22 cm fibula resected with the mass (Figure 2C). The popliteal vein was ligated and cut above the tumoral thrombus. We did not need vascular reconstruction or grafting since the saphenous veins and posterior and anterior tibial arteries were intact. All the other neurovascular structures were preserved. Early postoperative and follow-up examinations showed no neurovascular compromise. Patient mobilized immediately after the surgery with non-weight bearing for one week for soft tissue healing purposes.

**Figure 3:**
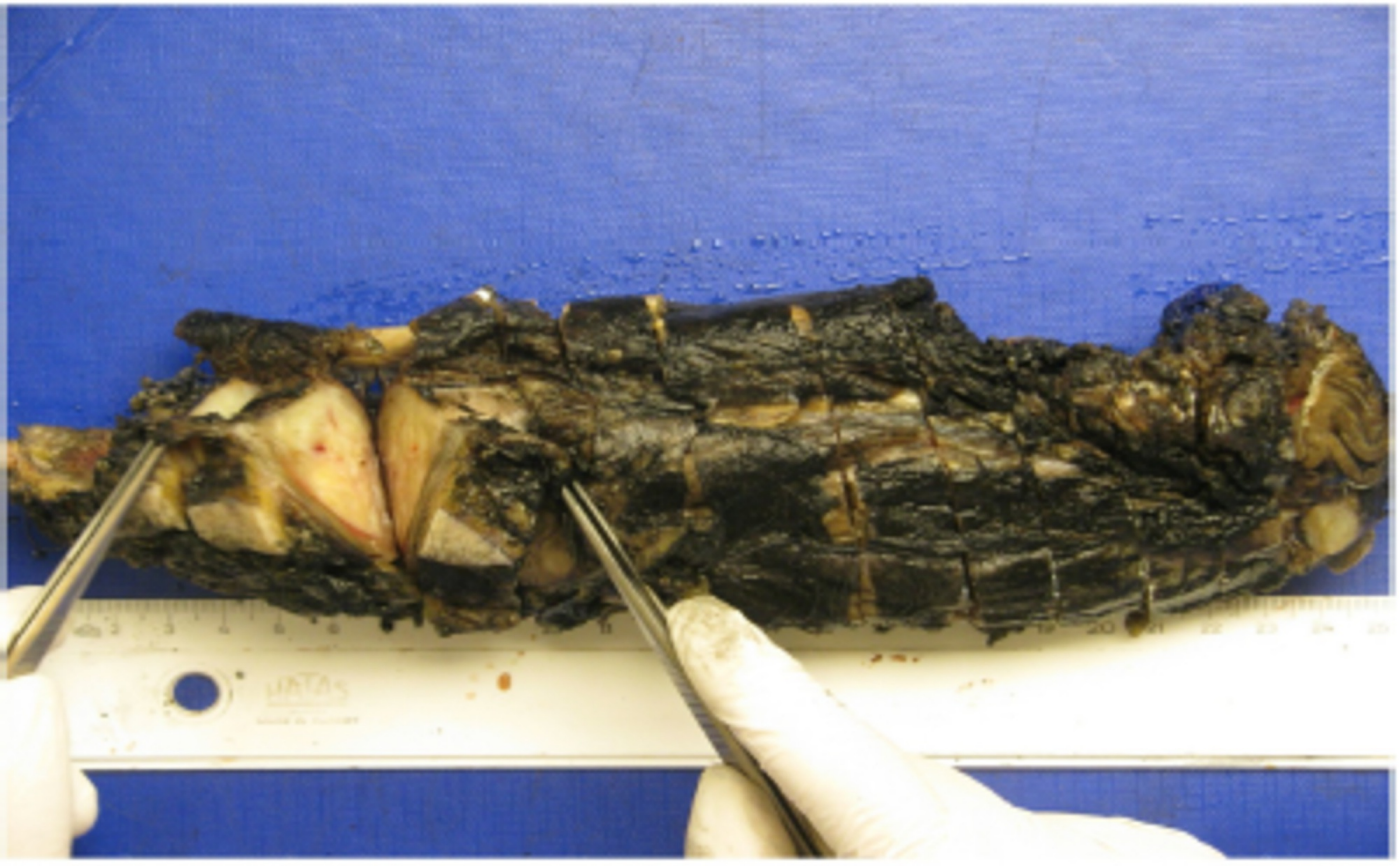
Macroscopic view of the specimen

The pathologic diagnosis was intravenous leiomyosarcoma (Actin, Alpha Smooth Muscle [1A4], SkyTek: positive, diffuse, strong; Caldesmon antibody [h-CALD], GeneTex: positive, diffuse, strong; CD34 antibody [QBend/10], SkyTek: negative; TLE1 antibody [polyclonal], GeneTex: negative) with intact surgical margins and 90% necrosis (Figure 3). A control abdominal PET-CT showed focal 18F-fluorodeoxyglucose take-up in the pancreatic head, which had no correlation with the CT. An endoscopy and MRI showed an 8 mm uncinal mass in the pancreatic head, which is also a very rare metastatic site for leiomyosarcomas. It was surgically excised. Adjuvant chemotherapy continued for six cycles. There was no recurrence in the six-month and one-year and eighteen-month follow-up radiological examinations. Written informed consent was obtained from the patient for this case study.

## Discussion

Leiomyosarcomas originating from vascular structures are rare, with the most common age of presentation in the sixth and seventh decades [1]. Most of these cases arise from the large veins, such as the inferior vena cava, external iliac vein, femoral vein, and popliteal vein. There are only two cases of leiomyosarcomas of the posterior tibial vein in the literature, and these originated from the veins distal to the popliteal vein [2]. To our knowledge, this is the first case of primary vLMS of the peroneal vein. The differential diagnosis includes spindle cell-shaped neoplasms such as benign and malignant tumors of the nerve sheaths, myofibroblastic tumors (myofibromatosis, fibromatosis, and myofibroblastic sarcoma), synovial sarcoma, and fibrosarcoma [3]. Leiomyosarcomas have a limited response to chemotherapy and radiotherapy. Surgical excision with wide margins is thus the treatment of choice [4]. Vascular structures with close contact can be dissected with the tunica adventitia left on the mass. This approach can prevent sacrification of the vascular structures. Frozen-section pathologic consultation should be included in this plan. Bone invasion is an indication for bone excision and reconstructive modalities. On the other hand, like adventitia, the periosteum is accepted as a good barrier. Tumors like extremity leiomyosarcomas can be dissected from bone subperiosteally with the periosteum left on the tumor. Reconstruction options include intercalary prosthesis, biological reconstruction, or reconstruction with liquid nitrogen-treated recycled bone. We were not in need of osseous reconstruction since the tumoral invasion was in the middle third of the shaft area of the fibula. Adjuvant radiotherapy is commonly used postoperatively, especially for tumors originating from neurovascularly tight sites such as the popliteal artery or the proximal thigh. Local recurrence rates are reportedly as high as 66% [5]. Adjuvant chemotherapy is indicated for metastatic disease. Intralesionary excision and an unresectable tumor site are indications for radiotherapy and chemotherapy alone. The pancreas, especially the head, is a rare metastatic site for all malignancies and extremely rare for vLMS [6]. Most metastatic lesions occur in the lungs and liver. In the case of intra-abdominal and pulmonary vascular tumors, the adjacent organs are at high risk for local invasion.

## Conclusions

Distal vLMSs are very rare but must be considered in the case of extremity soft tissue sarcomas, even among patients of a young age. The biopsy sites should be planned in accordance with the final surgical approach. Metastatic lesions are not rare for vLMS, so even if the site is unusual and the radiological findings inconsistent, all suspicious lesions should be accepted as metastasis until proven otherwise. Surgical excision with wide margins is the treatment of choice, and high rates of local recurrence should be kept in mind when planning the surgery.
